# Protocol for a randomized controlled clinical trial investigating the effectiveness of Fast muscle Activation and Stepping Training (FAST) for improving balance and mobility in sub-acute stroke

**DOI:** 10.1186/s12883-014-0187-y

**Published:** 2014-10-10

**Authors:** Kimberly J Miller, Michael A Hunt, Courtney L Pollock, Dianne Bryant, S Jayne Garland

**Affiliations:** The University of British Columbia, 212 Friedman Building, 2177 Wesbrook Mall, Vancouver, BC V6T 1Z3 Canada; School of Physical Therapy, Faculty of Health Sciences, The University of Western Ontario Elborn College, Rm 1438, London, ON N6G 1H1 UK

**Keywords:** Stroke, Postural control, Randomized clinical trial, Rehabilitation, Physiotherapy, Exercise therapy, Treatment outcome, Electromyography, Walking

## Abstract

**Background:**

Following stroke, many people have difficulty activating their paretic muscles quickly and with sufficient power to regain their balance by taking quick and effective steps. Reduced dynamic balance and mobility following stroke, or ‘walking balance’, is associated with reduced self-efficacy and restrictions in daily living activities, community integration, and quality of life. Targeted training of movement speeds required to effectively regain balance has been largely overlooked in post-stroke rehabilitation. The Fast muscle Activation and Stepping Training (FAST) program incorporates fast functional movements known to produce bursts of muscle activation essential for stepping and regaining standing balance effectively. The purpose of this study is to: 1) compare the effectiveness of an outpatient FAST program to an active control outpatient physiotherapy intervention in improving walking balance following stroke, and 2) explore potential mechanisms associated with improvements in walking balance.

**Methods/Design:**

This will be an assessor-blinded, parallel group randomized controlled trial design. Sixty participants (30 per group) who have sustained a stroke within the previous six months will be randomly assigned with stratification for lower limb motor recovery to receive twelve 45-minute 1:1 physiotherapy intervention sessions over 6 – 10 weeks in an outpatient setting of either: 1) FAST intervention - systematic and progressive practice of fast squatting and stepping exercises, or 2) active control - conventional physiotherapy directed at improving balance and mobility that includes no targeted fast movement training. The same blinded research physiotherapist will assess outcomes at three time points: 1) baseline (prior to intervention), 2) follow up (within one week post-intervention); and 3) retention (one month post-intervention). The primary outcome is dynamic balance assessed using the Community Balance and Mobility Scale. We will also assess fast and self-selected walking speed, balance self-efficacy, and the ability to respond to internal and external perturbations to balance and associated changes in postural muscle activation.

**Discussion:**

The targeted training of fast functional movements in the FAST program is expected to improve walking balance following stroke compared to the active control intervention. Unique to this study is the investigation of potential mechanisms associated with improvements in walking balance.

**Trial registration:**

NCT01573585

**Electronic supplementary material:**

The online version of this article (doi:10.1186/s12883-014-0187-y) contains supplementary material, which is available to authorized users.

## Background

Balance ability is one of the most important factors in determining independence in daily activities and risk of falls following stroke [1]. Dynamic balance and mobility or ‘walking balance’ can be described as the ability to control the centre of mass (COM) within the base of support to remain upright during ambulation [2] and encompasses a range of walking-related tasks which challenge the balance system. The impact of diminished dynamic balance can be significant for stroke survivors. The resulting mobility limitations are associated with decreased self-efficacy, loss of independence, and restrictions in activities of daily living, community integration, and quality of life [3,4].

People following stroke struggle to produce sufficient power (the product of force and speed) with their paretic muscles; deficits in speed of movement are well-documented after stroke [5,6]. It has been suggested that power may be more important than absolute force production in functional mobility tasks, and velocity of movement may be instrumental to counteract potentially destabilizing forces exerted on an individual’s COM by gravity and environmental interactions (external perturbations) as well as voluntary movements (internal perturbations) [7,8]. Modest perturbations to quiet stance can be accommodated using an inverted pendulum model of balance control [9] in which the ankle musculature plays a pivotal role in the maintenance of stance, while activation of muscles around the hips is essential in responding to larger perturbations [10,11], particularly if a step is required to maintain balance - termed a stepping reaction [12]. There may be a greater reliance on stepping behaviours to avoid a fall after stroke than in healthy individuals [13]; however, impairments in paretic muscle activation make it difficult for these individuals to step with sufficient speed, coordination and amplitude to effectively to regain their balance [7,14,15].

Current clinical practice guidelines advocate task-oriented exercise programs involving the practice of ‘real life tasks’ following stroke [16], but specificity of task practice with respect to the speed required for postural reactions has received relatively little attention. A single session of fast functional squat and stepping training in stroke survivors has been found to improve paretic muscle activation and postural responses during an internal perturbation balance task after the exercises [15,17]. A recent case series reported improvements in walking balance that were retained for one year following a 12-session stepping retraining program in community-dwelling individuals with chronic stroke [18]. These changes in balance were accompanied by an increase in participation in meaningful activities in the community, and improvements in the movement kinematics of stepping reactions. These findings suggest that Fast muscle Activation and Stepping Training (FAST) may be an effective approach to improve walking balance following stroke.

The primary objective of this assessor-blinded, parallel design, randomized controlled trial is to investigate the effectiveness of an outpatient FAST program commenced within the first six months following stroke compared to an active control intervention of conventional outpatient physiotherapy (active control) that includes no targeted fast movement training for improving walking balance. As many people fall in the first few months after discharge from inpatient rehabilitation, this was thought to be an important window for this balance intervention [19]. The primary hypothesis is that improvements in walking balance (as measured with the Community Balance and Mobility Scale, CB&M [20,21]) will be larger following 12 sessions of FAST intervention compared to 12 sessions of active control intervention. The secondary hypothesis is that CB&M scores will continue to be higher in the FAST group compared to the active control group one month following the intervention. Another aim of this study is to explore potential mechanisms associated with improvements in walking balance including; improvements in walking speed, balance self-efficacy, ability to respond to internal and external perturbations to balance and associated changes in postural muscle activation. Our *mechanistic hypotheses* are that compared to the active control intervention; the FAST retraining will result in:

- Increased fast and self-selected natural walking speed (gait assessment);
- Reduced postural sway (balance perturbation assessment);
- Improved timing and amplitude of muscle activation (EMG onset timing, burst area and slope) during balance perturbations and gait;
- Increased balance self-efficacy (using Activities-specific Balance Confidence (ABC) scale).

## Methods

### Study design

**Figure 1:**
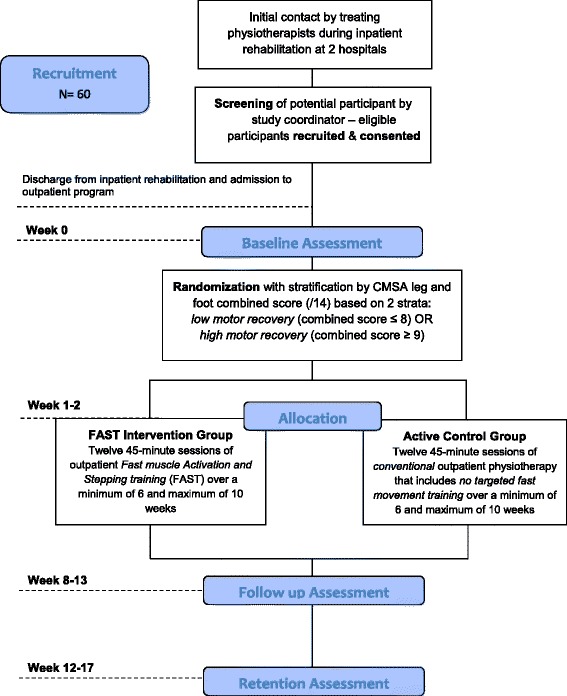
**Design of the FAST Study.** Flow chart illustrating study design from initial contact with potential participants to retention assessment. CMSA, Chedoke-McMaster Stroke Assessment.

The study is a single-blind, parallel design, randomized controlled trial, with the assessor blinded to the group allocation of the participants (Figure 1). The participants cannot be blinded to the interventions that they receive, though they will not be aware of the details of the other study arm. Eligible participants who provide written consent will be randomly assigned, to the FAST or active control group. Interventions will be received following discharge from inpatient rehabilitation in the outpatient departments of two hospitals in the Greater Vancouver area; Holy Family and Lion’s Gate Hospitals. Outcome measurements will be evaluated at the University of British Columbia at three time points (Figure 1): 1) baseline (prior to the first intervention session), 2) follow up (within one week of the 12th intervention session), 3) and retention (one month following the 12th intervention session) by the same blinded assessor. This study was approved by the University of British Columbia Human Research Ethics Committee. This protocol is reported in accordance with SPIRIT guidelines [22,23].

### Selection criteria and recruitment of participants

Individuals who have had their first stroke (confirmed by admission CT scan) within the previous 6-months that resulted in unilateral hemiparesis and required in-patient rehabilitation will be invited to participate if they also meet the following inclusion criteria:

- Sufficient motor control in the paretic lower limb to perform the stepping activities in the FAST program: Chedoke McMaster Stroke Assessment (CMSA) [24] leg and foot scored as stages 3–6 (stage 7 consider ‘normal’). CMSA leg and foot scores reflect the presence and severity of motor impairment following stroke, and they are used clinically to evaluate motor recovery [25].
- Standing balance ability necessary to participate safely in the FAST program: Berg Balance Score (BBS) ≥ 30/56 [26]. The BBS has established validity and excellent reliability in for assessing functional balance in people following stroke [27].
- Cognitive capacity to provide informed consent: Mini-Mental Status Examination score ≥ 24/30 [28].

We will exclude individuals with:

- Bilateral stroke or a history of previous stroke(s) for which inpatient rehabilitation was received.
- Severe co-morbidities likely to dominate the pattern of care (e.g. metastatic disease, severe congestive heart failure, etc.), co-existing peripheral neuropathies or vestibular disorders likely to independently have a negative impact on balance, and severe musculoskeletal problems or pain; as these conditions would be likely to impede participation in the study interventions.
- Global aphasia, receptive aphasia or language barriers who do not have someone to assist them in translating information, as these individuals would have difficulty providing informed consent or understanding exercise instructions.

The initial contact with potential participants will be made by the inpatient physiotherapist who is treating the potential participants at the two participating hospitals. The physiotherapist will ask them if they are interested in learning more about the study and, if interested, ask them if they will give consent to have the research coordinator review their chart to confirm eligibility. After reviewing their chart, the research coordinator will approach the prospective participant to describe the study in more detail or explain why they are not eligible. The research coordinator will review the letter of explanation and the consent form with the prospective participant and answer any questions. The research coordinator will return at least 24 hours later to obtain written consent before participating. Family members may be present to assist if there are any language barriers. All patients admitted with a primary diagnosis of stroke will be screened by their physiotherapists at two hospitals using a screening checklist. In accordance with CONSORT guidelines [29], the number of screened patients who are ineligible (and the reasons for ineligibility) or who are potentially eligible but are not interested in the study will be recorded.

To optimize recruitment, all members of the stroke rehabilitation teams at each recruitment site including physiotherapy managers, site coordinators, intake clerks and physiotherapists will be engaged in refining the processes for screening and recruitment specific to their organizations; strategies that are tailored to the sites will be developed collaboratively including reminders, flow charts for processes and communication, and screening forms designed to minimize burden for the site staff and their patients. These processes will be reviewed and revised based on feedback and will be sustained by onsite coordinators who serve as local leaders and champions for the study in collaboration with the research coordinator.

### Group allocation and data management

Data will be managed using web-based software (EmPower Health Research, http://www.empowerhealthresearch.ca/). Upon registration, the software assigns each participant a unique identification number. Upon screening eligibility, providing signed consent and completion of the baseline assessment, participants will be randomized by the research coordinator using the integrated randomization software, “Integration”. This software protects allocation concealment by ensuring screening is complete and the participant is eligible prior to randomization as well as preventing multiple allocation of the same participant or unjustified removal of participants post-randomization. Participants will be stratified by their (combined leg and foot – affected limb only) CMSA score (≤8 or ≥9) to force a balance between groups in the motor recovery of the leg and foot. The randomization scheme is set up in permuted blocks of 2 and 4 to ensure a similar number of participants between groups and to prevent screening clinicians from guessing the next allocation. All outcome data will be entered into EmPower by the research coordinator, and the accuracy of data entry will be double-checked by a second person who will be blinded to participant group allocation. Research personnel who are to remain blind to group allocation (outcome assessors, data analysts) will not have access to the randomization form when they log into the database to enter data. The data management software contains an audit log that records the user with date and time stamps for all data entries, changes to data and explanations for changes. Data verification and the audit trail ensure high standards of data integrity and validity.

### Intervention

Participants in both intervention groups will receive 12 individual 45-minute physiotherapy sessions. These sessions are intended to be scheduled twice per week over a 6 week time frame. A pragmatic window will be set for completion of the 12 sessions within 10 weeks to accommodate interruptions (e.g. illness, vacations, etc.). This dosage is established firstly, to be consistent with the estimated dosage of regular out-patient physiotherapy following stroke reported by the two participating hospitals, thereby supporting the potential external validity of the study, and secondly, based on previous research reporting improvements in community- level balance and mobility (assessed by CB&M score), and in movement kinematics following 12 sessions of stepping reaction training in individuals with chronic stroke [18].

### FAST intervention program

The FAST intervention will involve the systematic and progressive practice of fast functional movements to retrain the rapid bursts of muscle activation and anticipatory postural adjustments of the leg and trunk muscles essential for regaining standing balance and community mobility activities (Table 1) [7,15,17,30]. The treatment physiotherapist providing the training will apply motor learning principles, in particular, using the ‘challenge point framework’ [31], previously applied successfully to retrain stepping reactions in community-dwelling individuals following stroke [18]. Using this approach the conditions of practice including the organization of practice (e.g. blocked versus random practice schedules), the level of physical support (e.g. use of a overhead harness versus a walking belt with standby assistance of a therapist), and the feedback provided (e.g. immediate versus summary feedback) will be adjusted by the treatment physiotherapist and progressed according to the skill level of the performer and the relative difficulty of the task to reach optimal ‘challenge points’ for learning and skill transfer to balance disturbances in everyday life [18,31].

**Table 1:** **FAST intervention program content**

**1**	**Squats**
Squats to approximately 30 degrees of hip and knee flexion “as fast as possible” to promote a sudden braking action.	
Typical instruction: “*Unlock your knees and stop the downward movement as quickly as possible*.”	
	• Dosage: Work up to 5 sets of 10 reps. Allow approximately 5 s between each rep and 30 s (or longer) between each set
**2**	**Steps**
The core element of the FAST intervention, step training is to be included in every treatment session. Participants will lean, pivoting at their ankles until they need to take protective step(s) to stop themselves from falling. A typical instruction is provided (below); however, treatment physiotherapists will tailor instructions and feedback to the participant, based on their performance and abilities.	
Typical instruction: “*Lean [forward/backward/to the side] and let yourself fall like a tree until you feel like you are losing your balance. The goal is to take steps that are long enough and fast enough that you are able to regain your balance*.”	
Progressions of step activities are listed below in increasing level of difficulty:	
*a. Simple blocked practice*	
Stepping leading with each leg in each direction is practiced in blocks of 10 reps.	
	• Dosage: Work up to 2 sets of 60 reps. Each set consists of 10 reps each of leading with paretic (P) and non-paretic (NP) leg leaning forward and backward directions, and 10 reps each of lateral leaning to P and NP side (lead leg not specified).
*b. Semi-random practice:*	
	i. Stepping leading with each leg in each direction is practiced in blocks of 5 reps
	• Dosage: 5 sets of 5 reps with each leg (or to each side for the lateral leaning task) in each direction (75 reps with each leg/to each side in total)
	ii. Stepping using alternate leading legs/leaning side (P then NP) each time, for a total of 10 reps (5 reps/side) forward, backward and laterally.
	• Dosage: 5 sets of 10 reps (75 reps with each leg/to each side)
*c. Random practice:*	
Stepping leading with the P leg in all 3 directions - forward, backwards, and laterally to P side, then leading with the NP leg in all 3 directions.	
*d. Random nomination of lead leg with reduced movement planning time:*	
Therapist randomly nominates the lead leg after leaning in the forward or backward direction has been initiated by the participant.	
	• Dosage: Target of 20 steps with P leg in the forward and backward directions (40 reps total).
*e. Concurrent task planning:*	
Participants asked to perform a simple concurrent cognitive task (e.g. counting backwards from 10) as they lean and step. Start with the simple blocked practice (2a) and progress to random practice (2c).	
**3.**	**Complementary activities to add to challenge and interest:**
*a. * *Step over -4-square exercise:* Masking tape will be affixed to the floor in a ‘+’ design creating 4 squares. The participant will begin with both feet in a square facing forward, and move in a counterclockwise direction through each square in sequence until they reach the ‘start’ square; they will than move in a clockwise direction to again return to the ‘start’ square [32]. The following instructions are given to the patient ‘try to complete the sequence as fast as possible without touching the tape lines. Both feet must make contact with the floor in each square. If possible, face forward during the entire sequence.’ Timing and accuracy can be used as feedback.	
*Progress* – ask Participant to ‘bound’, rather than step over the pattern.	
*b. Additional stepping and bounding activities*	
	i. Leaning and stepping off a Sissel Balancefit dome (or similar support)
	ii. Stepping onto/off of a stable low block/step, progressing to bounding on/off block/step
	iii. Bounding off one leg to land in a step-to position working toward a ‘flight phase’ with both feet off the ground, progress to bounding and landing on the opposite foot.
For these exercises ‘shock absorption’ by the landing leg is to be emphasized (return to mini-squats to emphasize if necessary). Therapists will monitor to insure any pre-existing musculoskeletal symptoms are not aggravated. Activities will be practiced first in a forward direction, then progressing to lateral and then backwards directions; start with blocked practice (as in 2a), then progress to random practice schedule (as in 2c).	
	• Dosage: Work up to 60 repetitions with each leg/side

The content of the FAST intervention program is provided in Table 1. The progression of exercise tasks will be based on the clinical judgment of the treatment physiotherapist in conjunction with the participant [18]. The exercises will include:

1. *Squats*: This exercise was associated with short term improvements in muscle activation and postural responses to balance perturbations in individuals with chronic stroke [15,17]. Squats will be used as a warm up activity and to encourage more equal weight distribution between the paretic and non-paretic legs. As participants improve, this squatting task will be relatively easy to perform, and therefore more time within the intervention session will be spent performing the stepping activities.
2. *Stepping*: The progressive retraining of the quick automatic stepping responses required to re-establish balance following perturbations is the core element of the FAST intervention. The stepping training activities will be based on a previously published program used with community dwelling individuals following stroke, and adapted to the abilities of the subacute stroke participant group [18]. Initially, participants will be permitted to take as many additional steps as required to regain their balance, but will later be instructed to stop their momentum within 2–3 steps. This stepping activity elicits fast bursts of muscle activation in the stepping leg(s) and fast postural adjustments to alterations in balance [15]. Step exercises will be performed with both legs in all directions (forward, backward and laterally to each side).
3. *Complementary activities*: Finally, additional complementary activities will be provided to add challenge and variety for high level participants who quickly and easily complete the core step training program within the individual intervention sessions. These activities were selected as they were likely to evoke fast bursts of muscle activation and quick steps consistent with the goal of practicing fast lower limb movements. They will include the option of a 4-square exercise adapted from the clinical ‘4-square test’ of stepping and change of direction [32], and more challenging stepping and bounding [33] activities.

### Active control program

Similar to the FAST intervention, the active control intervention will include activities tailored to the individual needs and goals of the participant to regain balance and mobility [34]. The content of the active control physiotherapy intervention will include task-specific practice of standing balance and walking activities, as well as stretching, strengthening and endurance exercises. The main difference in the treatment between active control group and what might be delivered in regular out-patient physiotherapy is that there will be no specific training of fast stepping responses and no systematic training of fast over-ground walking (including treadmill training). These activities are not typically part of the conventional treatment provided at the two participating hospitals. Additionally, there will be no training of bounding, hopping or other exercises that emphasize high velocity contraction.

### Concomitant care

During the study, participants will be advised not to undertake concomitant specific balance and mobility training or treatment. Participants will receive their other outpatient treatment programs (e.g. occupational therapy, speech-language therapy) and can engage freely in community-based activities that may include aqua-exercise, senior’s activity programs, etc.

### Intervention fidelity and monitoring of adverse events

Treatment physiotherapists who will be delivering the FAST and active control interventions will be provided with a treatment handbook outlining the intervention program. This material will be reviewed in a group training session with case examples and opportunities for discussion to provide feedback to refine and clarify the written materials. During the study, the research coordinator, an experienced physiotherapist and clinical researcher, will go to individual sites to facilitate the implementation of the proposed interventions, providing 1:1 guidance specific to the knowledge and beliefs of the treatment physiotherapists, their environment, and the needs of their participants while still ensuring the critical elements of the intervention are provided as planned [35]. Standardized recording forms (Additional files 1 and 2) developed with feedback from the treatment physiotherapists will be used to record the content and duration of interventions provided to the participants. The number of repetitions of activities, the level of assistance (overhead harness, transfer belt), scheduling of practice (blocked, semi-random, random) and feedback (immediate, summary) will be recorded for each FAST intervention session.

Ongoing correspondence will be maintained with the treatment physiotherapists to ensure that the interventions are being provided as planned, to share information between treatment physiotherapists, and to further revise and clarify treatment information based on their feedback and experience. Audit and feedback based on the recorded treatment information will be used by the research coordinator to monitor and provide a post-hoc assessment of the FAST implementation fidelity in terms of primary measures of content (Table 1) and minimum dosage (indicated in ‘dosage’ for each activity, Table 1). The performance target is for all FAST participants to be capable of performing the random nomination of lead leg activity (Table 1, Activity 2d) with the support of a walking belt and standby assistance from a therapist by the end of the 12 sessions of intervention period. Similarly the content of the active control intervention will be audited and feedback will be provided in those instances where fast movement related interventions are documented. The individual onsite visits will be used to provide a formative assessment and feedback to the treatment physiotherapists regarding the extent to which the individual treatment sessions match with the intended intervention programs.

Participants entering the study will be warned that they may experience exercise-related muscle discomfort 24–48 hours following the intervention sessions, as many participants will not have participated in vigorous physical activities recently. Any unanticipated or adverse events will be documented in accordance with the policies of the hospital site and recorded in the EmPower data base, with referral for appropriate medical follow up. As this is an effectiveness study, the interventions will be modified to accommodate co-existing conditions (e.g. ankle sprain) and emerging issues (e.g. recovery from an acute respiratory infection).

### Outcome assessment and data collection

A summary of the measures to be collected at four time points (enrolment, baseline, follow up and retention) is provided in Table 2. The research coordinator will administer and record the MMSE to establish eligibility. The other clinical measures used to establish eligibility (BBS, CMSA leg and foot scores) and descriptive data including general demographic information (gender, age, hand dominance), stroke-specific information (date of stroke, type, side and location of stroke), co-morbidities and past medical history will be collected from charted information. Primary and secondary outcome measures and associated information recorded at Baseline, Follow up and Retention assessments will be conducted at University of British Columbia by the same blinded physiotherapy assessor. Participants will be asked to wear the same shoes for all assessment sessions.

**Table 2:** **Measures collected at enrolment and the three assessment time points**

	**Time 1: Study enrolment**	**Time 2: Baseline assessment**	**Time 3: Follow up assessment**	**Time 4: Retention assessment**
Demographic information	✓			
Stroke specific information	✓			
Medical information	✓			
Mini-mental Status Exam	✓			
Chedoke-McMaster Stroke Assessment leg & foot score	✓	✓	✓	✓
Berg Balance Scale	✓	✓	✓	✓
Community Balance and Mobility Scale		✓	✓	✓
Activities-specific Balance Confidence questionnaire		✓	✓	✓
10 Metre Walk Test (fast walking speed)		✓	✓	✓
Biodex – maximum voluntary contraction EMG		✓	✓	✓
Self-selected walking speed		✓	✓	✓
Gait – kinematic, kinetic and EMG parameters		✓	✓	✓
Physiological Balance Test – Postural stress test (external perturbations)		✓	✓	✓
Physiological Balance Test – Arm raise task (internal perturbations)		✓	✓	✓
Physiological Balance Test – Stepping Reactions (internal perturbations)		✓	✓	✓
Helpfulness of treatment received in improving balance			✓	✓

Reasons for missing outcome assessments or treatment sessions will be recorded. Where possible, outcomes will still be collected at planned time points, even if the treatment sessions were not completed, in accordance with intention-to-treat analyses.

### Primary outcome measure

#### Walking balance

The Community Balance and Mobility scale (CB&M) was selected as the primary outcome measure to evaluate change in walking balance [21]. The CB&M consists of 19 test items scored from 0 to 5 (with the exception of the stair descent item, scored/6) based on quality and/or speed of performance, for a maximum score of 96 with higher CB&M scores reflecting better balance and mobility performance. The CB&M demonstrates strong responsiveness in community-dwelling individuals following stroke relative to other commonly employed clinical measures of balance such as the BBS, which suffers from floor effects in this population [20].

### Secondary outcome measures

Secondary clinical and laboratory-based measures will be taken at all assessment sessions to explore mechanisms for improvements in walking balance including; improvements in fast and self-selected walking speed, balance self-efficacy, ability to respond to internal and external perturbations to balance and associated changes in postural muscle activation. Satisfaction with study interventions will also be evaluated.

#### Clinical and self-report measures

1. *Fast walking speed*: The average completion time over three trials of the 10 metre walk test will be used to determine ‘fast’ gait speed. Participants will be instructed to, “Walk as quickly as possible while still remaining safe” without a gait aid, if possible. Should participants require a gait aid, the same gait aid will be used in assessments at all time points. This measure has established test-retest reliability in individuals following stroke [36]. Gait speed provides an indication of functional mobility and has been shown to be sensitive to change in patients recovering from stroke [37].
2. *Self-reported confidence in balance activities:* The Activities-specific Balance Confidence Scale (ABC) [38] is a self-reported questionnaire requiring participants to rate their confidence in completing 16 activities that challenge balance in the community from 0 (no confidence) to 100 (very confident). This measure has strong discriminant validity in identifying individuals experiencing multiple falls following stroke [39].
3. *Satisfaction with physiotherapy interventions received during the study:* In response to the question: “Please rate the usefulness of the treatment that you received for improving your balance”, participants will rate interventions as 1 (not useful at all) to 5 (very useful) on a 5-point Likert scale.

#### Laboratory-based measures

Kinetics (force platform), kinematics (motion capture) and electromyography (EMG) will be collected in all the laboratory-based measures to investigate potential physiological mechanisms linked to functional changes following the FAST intervention. Kinetic, kinematic, and EMG data will be synchronized and collected simultaneously using commercially available software (Cortex, Motion Analysis Corp.).

### Data collection methods

Kinetic data will be collected using two floor-mounted force platforms (OR6-6, Advanced Mechanical Technologies Inc.) sampling at 2000 Hz. Kinematic data will be collected from 10 high-speed, high-resolution digital cameras (Raptor-E, Motion Analysis Corp.) that sample the movement of 22 reflective markers at 100 Hz. These reflective markers will be affixed to the participant’s skin according to a modified Helen Hayes marker set [40] bilaterally over the acromion processes, lateral epicondyles of the elbows, radial styloid processes, anterior superior iliac spines, lateral thighs, lateral femoral epicondyles, lateral tibiae, lateral malleoli, posterior calcanei, and on the ventral aspect of the feet over the bases of the 2nd metatarsal. A single marker will be placed over the sacrum. EMG data (Trigno^™^ Wireless EMG, Delsys Inc.) will collected at 2000 Hz by wireless surface electrodes placed bilaterally over the erector spinae, biceps femoris, quadriceps femoris, soleus and tibialis anterior muscles. The EMG data for each muscle group will be normalized to the maximum EMG recorded during three 5 s maximal voluntary isometric contractions performed while the participants are seated and secured onto the Biodex Isokinetic dynamometer (Model 900–860, Biodex Medical Systems, Inc.) using a methodology adapted from Hsu et al. [41]. The follow laboratory-based measures will be performed:

1. *Self-selected walking speed:* Participants will complete five walking trials at a self-selected natural pace along a 10 m walkway. Whole body COM will be calculated throughout the trial based on the positions of the reflective markers and using published anthropometric calculations [42]. Average walking speed from each trial will be calculated over the middle 4 m of the walkway based on the calculated movement of the whole body COM separately for the paretic and non-paretic lower limb. The overall self-selected gait speed will be obtained by averaging across the five trials [43].
2. *Physiological Balance Assessment:* Participants will be fitted into a safety harness attached to the ceiling and will stand (without assistive devices) with each foot on a separate force platform during the performance of the balance assessment tasks described below. Foot position on the platform will be standardized between outcome testing sessions using the tracings of each foot on paper adhered to the platforms. Participants will perform the following assessments:
  - *Quiet stance:* Participants will be instructed to look at a visual target and “stand as still as you can”. Five 10 s trials will be recorded to determine resting baseline EMG activation levels for each muscle.
  - *Limits of stability:* Participants will be asked to perform two maximal forward leans without raising their heels, hold the forward lean position for 5 s, and then return to their start position [44].
  - *External perturbations:* A Postural Stress Test adapted from Wolfson et al. [45] will be performed. Participants will stand with their eyes open focusing on a visual target at eye level while a series of forward perturbation forces (1, 2, 3, 4, and 5% of body weight) are exerted via a horizontal cable attached to the participant’s hips using a pulley system hidden behind a curtain. Using this paradigm, it will be difficult for participants to anticipate the timing of the perturbations. Participants will be instructed to “remain standing in place on the platforms.” The onset of the load drop will be measured using an accelerometer attached to the load being dropped. The highest load tolerated without taking a step will be repeated 5 times [46].
  - *Internal Perturbations:* Participants will be instructed: “When you are ready, raise your arm as fast as you can to shoulder height and hold it there” as they focus on a visual target at eye level [11]. Participants will perform 2–3 practice trials to familiarize themselves with the task and to receive any necessary correction on their performance. Ten trials with rest periods of 3–5 s between trials will be recorded. Arm acceleration magnitude will be measured using an accelerometer secured on the non-paretic hand to quantify the magnitude of the perturbation.
  - *Stepping reactions:* To evaluate change in stepping performance, participants will be asked to perform 5 blocked trials with each leg as the lead stepping leg in the forward direction [15,47].

### Analysis of mechanistic outcomes

The mechanistic outcomes from all the physiological balance tests will be the EMG characteristics (burst slope, burst area and onset timing [11,17,48]) and displacement of the whole body COM with respect to the centre of pressure (CP) including postural sway. The calculation of the COM using the motion capture software provides a measure of whole body movement. During feet-in-place tasks, CP displacement is representative of the body’s active control of balance which aims to maintain the COM safely within the base of support provided by the feet [49]. EMG onset timing, burst slope and area reflect the speed and magnitude of activation of postural muscles in anticipation of, or in response to balance perturbations.

1. *Centre of mass-centre of pressure (COM-CP) displacement*: The resultant ground reaction force data will be used to calculate the CP. The antero-posterior and medio-lateral coordinates of the COM position and the CP will be calculated for each data sample and used to calculate the resultant distance between the COM and CP in the transverse plane (COM-CP displacement). We will identify the maximum COM-CP displacement during each trial for physiological balance activities 2 *b-e.* These parameters will also be used to quantify postural sway in quiet stance (activity *2a).*
2. *Timing of EMG onset:* The onset of muscle activation burst (in milliseconds) will be calculated relative to the onset of the arm acceleration in the internal perturbations, the onset of load release for external perturbations, and onset of knee movement of the stepping leg for stepping reactions [15] for each trial.
3. *EMG burst slope and area:* The EMG burst area will be calculated separately for each trial. The EMG burst slope will be calculated from the average of the repeated trials. These parameters will reflect the timing and magnitude of the burst above quiet stance state and will be normalized to maximum EMG recorded on the Biodex.

### Sample size

Data from our published study of a similar FAST training protocol in individuals with chronic stroke showed an effect size of 1.9 SD with the intervention group and a pooled standard deviation of 7.45 for the pre and post CB&M score (primary outcome measure) [18]. Using our pilot data and a sample size equation suitable for our planned between-group comparisons using an analysis of covariance (ANCOVA); where we estimated that the magnitude of the association between the pre- and the post-intervention CB&M scores would be at least 0.75 and that a conservative estimate of effect size would be 0.60, we estimate that 30 participants per group (60 participants in total) are required to maintain a Type I error rate of 5% and 80% power to detect a difference between intervention groups. Therefore, recruitment will continue until a total of 30 participants in each group have completed the follow up assessment.

### Statistical analyses

The primary analysis will follow the intention to treat (ITT) principle; such that, once the participants are randomized they will be analyzed within their randomly assigned group, regardless of compliance with the protocol. As part of the ITT analysis, participants will not be removed from the analysis unless it can be shown that the participant was ineligible prior to randomization (and therefore should never have been included).

#### Primary analysis

We will perform an analysis of covariance (ANCOVA), where the baseline CB&M score serves as the covariate; treatment group as the independent variable, and the follow up CB&M score as the dependent variable. To test our secondary hypothesis about retention, we will repeat this analysis with the retention CB&M score as the dependent variable.

### Analyses of mechanistic outcomes

We will conduct a similar analysis for all other continuous outcome variables (maximal and self-selected walking speed, EMG parameters, and COM-CP displacement, and ABC score). Because our outcomes are related, we will not adjust our alpha error rate, but rather, look for consistency across outcomes. We will conduct an exploratory analysis to determine whether the variability in walking balance can be explained by the initial participant characteristics (side of lesion, type of stroke, CMSA score, initial ABC score, BBS score). To conduct these analyses, we will use a step-wise least squares method of linear regression where walking balance is the dependent variable, the pre-test measurement is a covariate, and group is an independent variable.

#### Dissemination plan

One of the collaborators in this study is a Knowledge Broker, hired by the Department of Physical Therapy to enhance knowledge translation. The Knowledge Broker will augment the uptake of the research findings from this current study in the clinical community. In addition to publishing the results in high quality journals and presenting findings at national and international conferences, the results will be disseminated through the funding agency, The Heart and Stroke Foundation of BC and Yukon, on their website. The authors also intend to disseminate the information through talks to local organizations including Stroke Recovery groups and will make the FAST training program freely available for download from the laboratory website.

## Discussion

The goal of this study is to determine whether an individualized physiotherapy exercise program geared to improving the speed of muscle activation (FAST) and incorporating motor learning principles is effective in retraining walking balance in an outpatient population following stroke. If effective, this novel exercise approach has the potential to significantly improve the rehabilitation strategies for walking balance, an activity which is of paramount importance for individuals following stroke. The study design and the intervention parameters have been deliberately established to optimize the generalizability the FAST program to other outpatient stroke clinical settings. Unique to this study is the focus not only on clinical effectiveness, but also mechanistic outcomes to evaluate physiological changes in walking and balance performance as well as perceived balance self-efficacy.

## Additional files

Additional file 1:
**FAST Treatment Record.** Standardized recording form to be used by treatment physiotherapist to document the actual treatment time, content, repetitions of activities, the level of assistance and feedback provided for each FAST intervention session. Additional file 2:
**Active Control Treatment Record.** Standardized recording form to be used by treatment physiotherapist to document actual treatment time, and content of Active Control intervention sessions.

## Abbreviations

FAST: Fast muscle Activation and Stepping Training
CB&M: Community Balance and Mobility Scale
COM: centre of mass
COP: centre of pressure
ABC: Activities-specific Balance Confidence scale
CMSA: Chedoke-McMaster Stroke Assessment
BBS: Berg Balance Scale
EMG: electromyography
ANCOVA: analysis of covariance
