# Supplementary material for: Protocol for a randomized controlled clinical trial investigating the effectiveness of Fast muscle Activation and Stepping Training (FAST) for improving balance and mobility in sub-acute stroke
Source: BMC Neurol. 2014 Oct 10;14:187. doi: 10.1186/s12883-014-0187-y (PMC4207320; doi:10.1186/s12883-014-0187-y)
Supplement: Additional file 1: — FAST Treatment Record. Standardized recording form to be used by treatment physiotherapist to document the actual treatment time, content, repetitions of activities, the level of assistance and feedback provided for each FAST intervention session. [file 12883_2014_187_MOESM1_ESM.docx]

Response to previous treatment session:

| **Exercise** | **Support**  H- harness;  B-transfer belt;  S-standby assist | **Practice time**  (to nearest 5 min) | **Dosage** (reps/sets)  Specify (H) for reps by hemiparetic leg | **Challenge variation(s)** | | | **Other relevant info** |
| --- | --- | --- | --- | --- | --- | --- | --- |
|  |  |  |  | **Practice** *Specify # where appropriate*  Blocked (B)  Semi-random(S)  Random (R) | **Task demand**  *Specify combo where appropriate*  Distance (d)  Time (t)  Random nomination (r)  Other | **Feedback**  *Specify combo where appropriate*  Verbal (V) +  (s) summary /(i) immediate  Support by therapist req’d (W)  Time limitation (T)  Other |  |
|  |  |  |  |  |  |  |  |
|  |  |  |  |  |  |  |  |
|  |  |  |  |  |  |  |  |
|  |  |  |  |  |  |  |  |
|  |  |  |  |  |  |  |  |
|  |  |  |  |  |  |  |  |
|  |  |  |  |  |  |  |  |
|  |  |  |  |  |  |  |  |
|  |  |  |  |  |  |  |  |
|  |  |  |  |  |  |  |  |

Response to treatment:
