# Supplementary material for: Protocol for a randomized controlled clinical trial investigating the effectiveness of Fast muscle Activation and Stepping Training (FAST) for improving balance and mobility in sub-acute stroke
Source: BMC Neurol. 2014 Oct 10;14:187. doi: 10.1186/s12883-014-0187-y (PMC4207320; doi:10.1186/s12883-014-0187-y)
Supplement: Additional file 2: — Active Control Treatment Record. Standardized recording form to be used by treatment physiotherapist to document actual treatment time, and content of Active Control intervention sessions. [file 12883_2014_187_MOESM2_ESM.docx]

Your patient is in the Active Control group of the FAST study. They should receive twelve 45-minute 1:1 physiotherapy sessions twice per week for a minimum of 6 weeks and maximum of 10 weeks (contact Research Coordinator if the frequency is consistently dropping to once/week). Please document the approximate time that you spend addressing the following categories of activities with your patient during each therapy session. This information is very important for interpreting the results of the study.

| **Date** | Session | Time (to the nearest 5 min) engaged in activity | | | | | | | | | |
| --- | --- | --- | --- | --- | --- | --- | --- | --- | --- | --- | --- |
|  |  | **Standing balance training (internal perturbations)**  UL activities, head/body rotation  **Provide a brief description* | **Standing balance training (changing BOS, external perturbations)**  stepping, tandem stand, (foam)  **Provide a brief description* | **Walking activities**  **Provide a brief description* | **Stair, obstacle activities** | **Other gross motor activities**  Sit-stand, sitting balance | **LE muscle strengthening** | **LE muscle stretching/**  **spasticity management** | **UE Activities/Rx** (not in standing) | **Other Rx activities** | Comments |
|  | 1 |  |  |  |  |  |  |  |  |  |  |
|  | 2 |  |  |  |  |  |  |  |  |  |  |
|  | 3 |  |  |  |  |  |  |  |  |  |  |
|  | 4 |  |  |  |  |  |  |  |  |  |  |
|  |  | **Standing balance training (internal perturbations)**  UL activities, head/body rotation  **Provide a brief description* | **Standing balance training (changing BOS, external perturbations)**  stepping, tandem stand, (foam)  **Provide a brief description* | **Walking activities**  **Provide a brief description* | **Stair, obstacle activities** | **Other gross motor activities**  Sit-stand, sitting balance | **LE muscle strengthening** | **LE muscle stretching/**  **spasticity management** | **UE Activities/Rx** (not in standing) | **Other Rx activities** | Comments |
|  | 5 |  |  |  |  |  |  |  |  |  |  |
|  | 6 |  |  |  |  |  |  |  |  |  |  |
|  | 7 |  |  |  |  |  |  |  |  |  |  |
|  | 8 |  |  |  |  |  |  |  |  |  |  |
|  |  | **Standing balance training (internal perturbations)**  UL activities, head/body rotation  **Provide a brief description* | **Standing balance training (changing BOS, external perturbations)**  stepping, tandem stand, (foam)  **Provide a brief description* | **Walking activities**  **Provide a brief description* | **Stair, obstacle activities** | **Other gross motor activities**  Sit-stand, sitting balance | **LE muscle strengthening** | **LE muscle stretching/**  **spasticity management** | **UE Activities/Rx** (not in standing) | **Other Rx activities** | Comments |
|  | 9 |  |  |  |  |  |  |  |  |  |  |
|  | 10 |  |  |  |  |  |  |  |  |  |  |
|  | 11 |  |  |  |  |  |  |  |  |  |  |
|  | 12 |  |  |  |  |  |  |  |  |  |  |
